# Spatial Regulation of Exocytic Site and Vesicle Mobilization by the Actin Cytoskeleton

**DOI:** 10.1371/journal.pone.0029162

**Published:** 2011-12-14

**Authors:** Jie Wang, David A. Richards

**Affiliations:** Department of Anesthesia, Cincinnati Children's Hospital Medical Center, Cincinnati, Ohio, United States of America; University of Massachusetts Amherst, United States of America

## Abstract

Numerous studies indicate a role for the actin cytoskeleton in secretion. Here, we have used evanescent wave and widefield fluorescence microscopy to study the involvement of the actin cytoskeleton in secretion from PC12 cells. Secretion was assayed as loss of ANF-EmGFP in widefield mode. Under control conditions, depolarization induced secretion showed two phases: an initial rapid rate of loss of vesicular cargo (tau = 1.4 s), followed by a slower, sustained drop in fluorescence (tau = 34.1 s). Pretreatment with Latrunculin A changed the kinetics to a single exponential, slightly faster than the fast component of control cells (1.2 s). Evanescent wave microscopy allowed us to examine this at the level of individual events, and revealed equivalent changes in the rates of vesicular arrival at the plasma membrane immediately following and during the sustained phase of release. Co-transfection of mCherry labeled β-actin and ANF-EmGFP demonstrated that sites of exocytosis had an inverse relationship with sites of actin enrichment. Disruption of visualized actin at the membrane resulted in the loss of specificity of exocytic site.

## Introduction

Although the core elements of the secretory machine are increasingly becoming understood at a molecular level, our understanding of their integration with the rest of the cellular architecture remains incomplete. This includes important questions such as ‘how do vesicles arrive at the plasma membrane’ and ‘how is this fusion directed to specific sites?’ Specificity of fusion is provided to some extent by the complement of cognate Q-and R-SNAREs found on the vesicular and plasma membranes, but this does not provide an explanation for the existence of exocytic hotspots found on the surface of neuroendocrine cells [1]. Equally, while the involvement of the cytoskeleton in secretion is clear, the complexity of that involvement has not been fully elucidated.

There is now extensive evidence that the actin cytoskeleton plays a role in exocytosis, both at fast synapses[2]–[4] and in neuroendocrine cells [5]–[12]. In electron micrographs of deep-etched freeze-fractured chromaffin cells, a lattice of actin filaments running parallel to the plasma membrane was observed, with clear attachment of the filaments to secretory vesicles [5]. This and other studies [6]–[7] led to the hypothesis that the actin cytoskeleton formed a barrier to secretion located just within the plasma membrane. Vitale and colleagues [6] showed that vesicles were excluded from a depth of approximately 50 nm inside the plasma membrane, and that stimulation allowed vesicles to move closer to the plasma membrane. This provided a simple mechanism for two pools of releasable vesicles in these cells; a readily releasable pool comprising vesicles already present at the plasma membrane, and a reserve pool of vesicles which are recruited upon depletion of the first pool. This model has required re-evaluation in the light of more recent findings that actin may influence the exocytic event itself [9], [11].

Actin dynamics have also been studied in fast synapses, where secretion is mediated by small synaptic vesicles (SSVs). Disruption of the actin cytoskeleton in these systems has been shown to result in an increase in release [3] or an impairment in vesicle mobilization [4]. As with dense-core vesicles, this may indicate that the cytoskeleton provides both transport and barrier functions.

PC12 cells have been extensively used as a model system for the study of secretion of large dense-cored vesicles (LDCVs), because of their ease of handling and consistency (see for example ref 7). We have used PC12 cells transfected with a releasable fluorescent cargo, ANF-EmGFP [13] to study secretion in cells co-expressing fluorescent actin, with and without disruption of the cytoskeleton using the G-actin sequestering toxin Latrunculin A. The goal of this study was to revisit previous findings on the influence of actin sequestering agents on burst and phasic release, using the technique of evanescent wave microscopy to study this process at the level of individual granules. This technique also allowed us to examine spatial aspects of this process, revealing that disruption of the actin cytoskeleton provided more available sites for exocytosis.

## Results

### Labeling the actin cytoskeleton in PC12 cells

**Figure 1:**
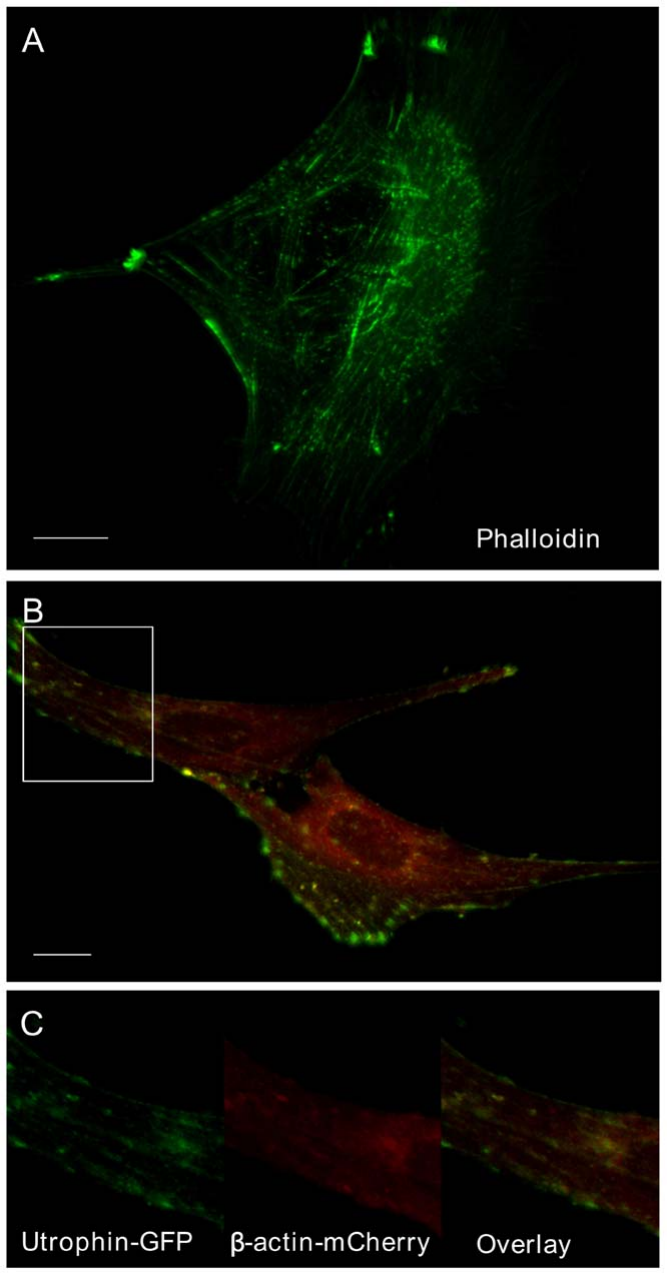
Comparison of staining with phalloidin, GFP-utrophin, and β-actin mCherry. (A) Fixed cell stained with alexa-fluor phalloidin. Note prominent staining of thick actin filamenents, while thin filaments are lightly stained. (B) Live cell, co-expressing Utrophin-GFP and β-actin -mCherry. Utrophin which only binds to fully stable actin filaments shows similar staining to that seen with phalloidin. β-actin mCherry labels all actin (monomer and filamentous, stable and dynamic). Area marked by white rectangle is shown in (C) at higher magnification, with the channels shown separated and overlaid.

In figure 1 three different means of labeling the actin cytoskeleton are compared; phalloidin-Alexafluor488, utrophin-GFP [14], and β-actin-mCherry. Phalloidin is not cell permeant, and so requires either fixation and permeabilization, or injection into the cell – it also strongly perturbs actin dynamics [15]. Utrophin-GFP and β-actin -mCherry both can be expressed and visualized in living cells, and label different sets of actin. Utrophin-GFP specifically binds to stable actin filaments much like phalloidin (Fig. 1A,B), which provides better signal to noise than β-actin -mCherry since it does not include any monomer labeling, but nor does it label the dynamic filaments that make up the connections to the plasma membrane. β-actin -mCherry, by virtue of its intrinsic fluorescence, does label all compartments; the disadvantage (shown in Fig. 1B,C) is that the filaments are less clearly distinguishable from the cellular background.

### Release of ANF-EmGFP from PC12 cells

**Figure 2:**
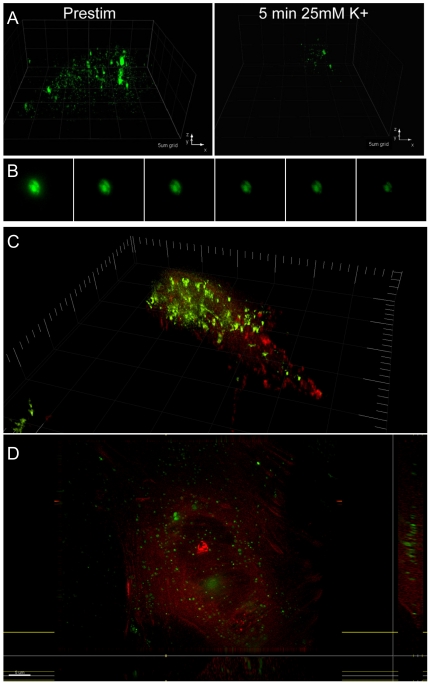
Release of ANF-EmGFP, and localization within the actin cytoskeleton. (A) 3D reconstruction of PC12 cell expressing ANF-EmGFP, before and after stimulation. Voxel dimensions are 108×108×200 nm. (B) Timelapse series showing a cell expressing ANF-EmGFP taken at 20 s intervals. (C) 3D reconstruction of PC12 cell co-expressing ANF-EmGFP and β-actin -mCherry. (D) Extended focus view of cell co-expressing ANF-EmGFP and β-actin -mCherry.

PC12 cells were transiently transfected with pre-proANF-EmGFP [13]. A three dimensional reconstruction of a typical cell is shown in figure 2A, showing fluorescent puncta distributed throughout the cell. On stimulation for 5 min with 25 mM high potassium ringer, the majority of the fluorescence had left the cell. In Fig. 2B we show a timelapse widefield image of a PC12 cell taken with a 20X objective (thus with a thick optical section), showing the rate of fluorescence loss.

Fig. 2C shows a three dimensional reconstruction of a living cell co-expressing the ANF-EmGFP vector, and β-actin cloned into the pmCherry vector (Clontech). In figure 1D a different cell expressing the same vectors is shown, using an extended focus view of the bottom 2 µm of the cell. This allows the structural information present in the red channel to be clearly seen. The staining pattern from the β-actin mCherry illustrates that the meshwork of actin filaments includes the majority of the cytoplasm, rather than just a narrow cortex around the plasma membrane, as is seen in harvested chromaffin cells [12].

### Latrunculin speeds the intial phase of secretion, while slowing the second phase

**Figure 3:**
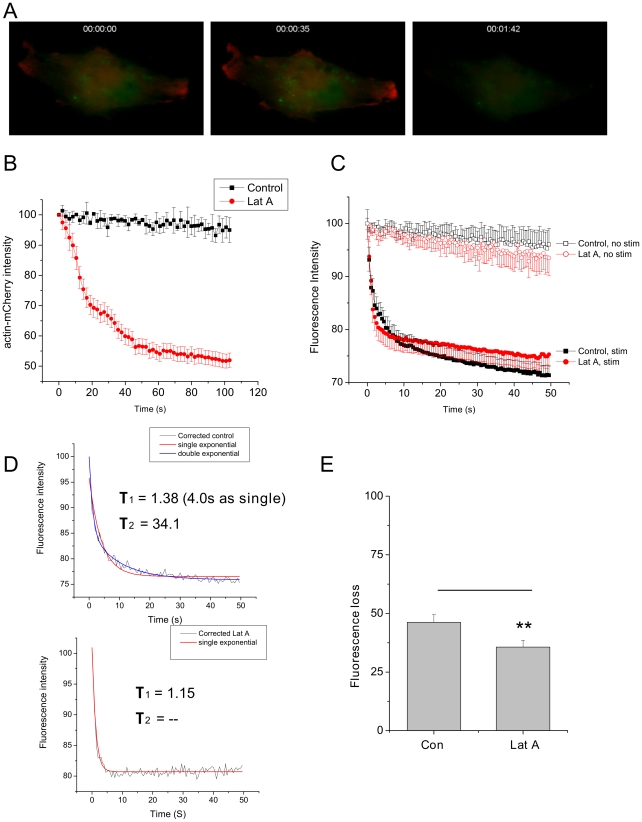
Effect of Latrunculin A on secretion from PC12 cells (A) PC12 cell co-expressing ANF-EmGFP and β-actin -mCherry. Times indicated reflect time after latrunculin addition. (B) Pooled data from 8 cells, measuring fluorescence intensity within a region corresponding to the outer 25% of the cell, in the presence (red) and absence (black)of Latrunculin A. Data are mean ± sem. (C) Rate of fluorescence loss, corresponding to secretion of ANF-EmGFP, in cells with (solid symbol) and without (open symbol) stimulation. In control cells (black) showed a biphasic release curve that could be fitted to a double exponential. Treatment with latrunculin A (red) caused opposite effects on the two phases of release; speeding the initial component, and slowing the second. Data are from 12 cells, mean ± sem. (D) If unstimulated curves were subtracted from the data corrected curves can be obtained. If these are then fitted, tau values for exponential fits are as shown. Control cells do not fit well to a single exponential (red), but are well described by a double exponential (blue). Latrunculin treated cells (lower) on the other hand are well fit by a single exponential (red). Unsubtracted numbers are Con; τ1 = 2.8 s, τ2 = 38.1 s; Lat A τ1 = 2.2 s, τ2 = 168.8 s. (E) Amplitude of fluorescence loss was calculated for 8 cells which were imaged twice; once before, and again after 2 min of stimulation. Latrunculin A caused a significant reduction (p<0.01) in the amount of fluorescence loss due to stimulation.

The ability to monitor the actin cytoskeleton in living cells with β-actin mCherry allowed us to directly measure the rate of action of latrunculin A, a commonly used actin sequestering agent derived from a Dead Sea sponge [16], on cells in our imaging chamber. In Fig. 3A, we show a sequence of three images over 100 sec following addition of 15 µM Latrunculin A. Especially at the flattened margins of the cell, a pronounced loss of red fluorescence was observed, corresponding to disruption of the cytoskeleton in these regions. If a region is created corresponding to the outer 25% of the cell, and fluorescence intensity is measured within this region, the timecourse of latrunculin action on the distribution of mCherry fluorescence can be measured (see Fig. 3B).

Having an indicator of the time course of latrunculin action, we next examined the effect of latrunculin on global secretion from PC12 cells. Following either 5 min incubation with 15 µM Latrunculin A, or the same period in normal ringer, cells expressing ANF-EmGFP were stimulated with 25 mM high potassium ringer. In untreated cells, release of ANF-EmGFP showed a biphasic release curve (Fig. 3C,D) that could be fitted to a double exponential. Treatment with latrunculin A caused opposite effects on the two phases of release; speeding the initial component, and slowing the second to the extent that the curve was best fitted to a single exponential (the slow component to a double exponential fit was 10 times slower than the experimental data). If cells with and without latrunculin treatment were stimulated for 2 min with images obtained before and after stimulation, latrunculin treatment was seen to inhibit overall release (Fig. 3E).

### Effect of latrunculin on rates of vesicle arrival and fusion

**Figure 4:**
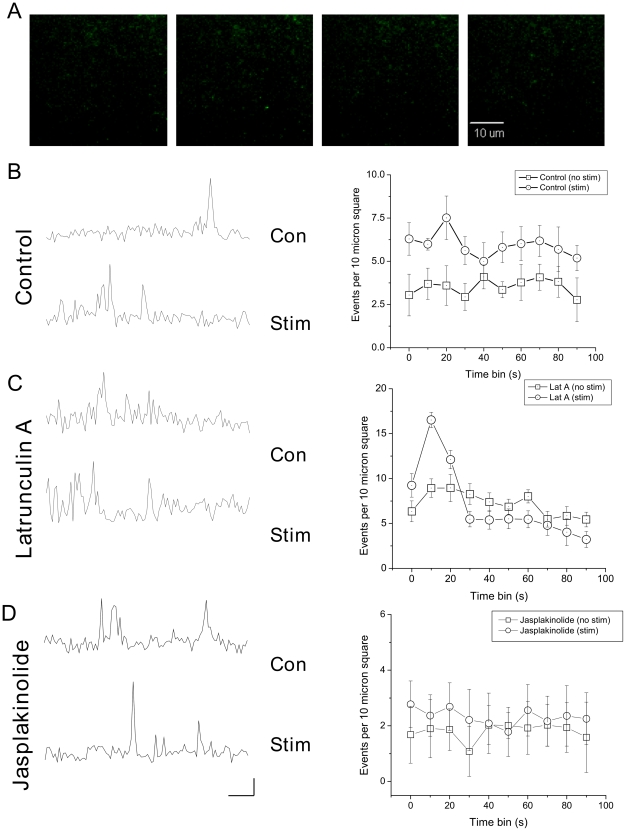
Effect of latrunculin and jasplakinolide on vesicle recruitment and secretion. (A) Visualized ANF-EmGFP vesicles can be observed arriving and fusing at the plasma membrane. (B) To the left, example ROI traces are shown before and after depolarization with 30 mM K+. To the right, release from 6 cells was analyzed before and after stimulation. Release was normalized as events per 10 µm^2^, per 10 s. (C) 6 cells were treated with latrunculin A; example ROI traces are shown to the left, and pooled data to the right. Latrunculin treatment caused an increase in spontaneous vesicular secretion; stimulation increased this further, however, this additional release fatigued rapidly (right). (D) 7 cells were treated with Jasplakinolide (10 µM), again, example ROI traces are shown on the left, and time binned averages on the right. Jasplakinolide treatment slightly reduced spontaneous rates of exocytosis, and prevented any significant increase following stimulation. Scale bar shows 10 s/10 arbitrary fluorescence units (directly comparable between conditions).

In order to gain better understanding of the interaction between the actin cytoskeleton and secretion, we took advantage of evanescent wave microscopy, also known as Total Internal Reflection Fluorescence Microscopy (TIRFM), which restricts fluorescent excitation to a small layer, approximately 100 nm deep, immediately above the cover glass. With this technique, individual vesicles labeled with ANF-EmGFP can be resolved [13], [17], [18] as small spots of fluorescence that appear and then disappear (Fig. 4A). Vesicles were seen to favor discrete sites of secretion. Under basal conditions (normal ringer) vesicles arrived at the plasma membrane at a steady rate. Stimulation with 30 mM Potassium enhanced the rate of secretion above this level (Fig. 4B). Treatment with latrunculin A caused an increase in the basal rate of secretion (Fig. 4C). Stimulation caused an immediate increase in secretion; however this was not sustained as steadily as in controls. If control and treated cells are compared, one can see that at the second time bin, the proportional initial stimulation was similar between the two groups, however, Latrunculin A treated cells showed rapid fatigue in later stages of secretion, declining even below unstimulated levels. Treatment with Jasplakinolide, a cell permeant actin stabilizing agent [19], slowed the rate of spontaneous secretion, while also preventing stimulation evoked release (Fig. 4D).

### Stimulation and latrunculin treatment differentially affect the rate of content expulsion

**Figure 5:**
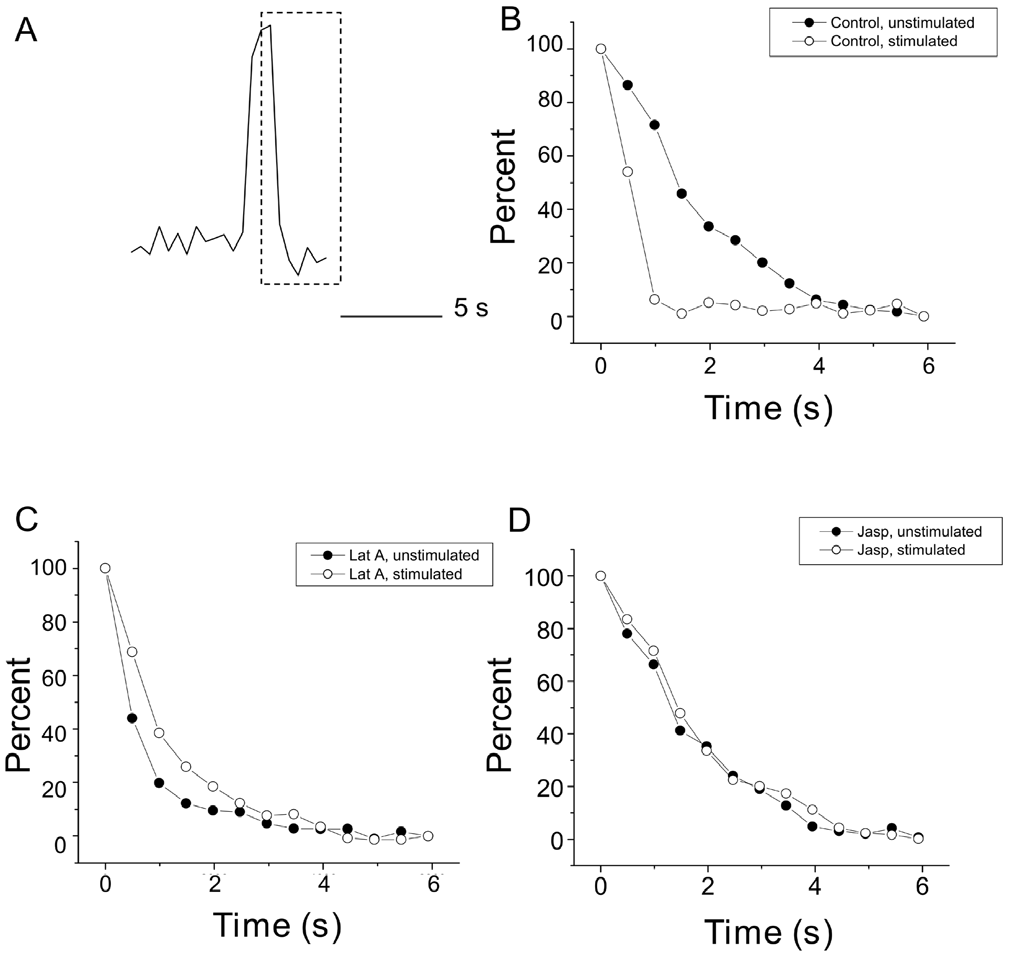
Actin facilitates expulsion of vesicular contents. (A) Example of individual exocytic event for vesicles labeled with either ANF-EmGFP, or Mepacrine, with the falling phase highlighted by the dashed box. (B) 50 events from 5 cells before and after stimulation were randomly chosen, based only on the criterion that they be clearly distinguished and separate from neighboring events (which tends to select for larger amplitude events also). The decay phase was aligned and averaged and is plotted here. Stimulation caused a marked enhancement of the rate of content expulsion. In latrunculin treated cells (C), when the same analysis was performed, the rate of content release was faster in unstimulated cells. (D) After jasplakinolide treatment the difference between unstimulated and stimulated rates of content expulsion disappeared.

In order to examine a potential role for actin in the exocytic reaction itself, we have examined the rate of loss of vesicular content before and after stimulation, and in the presence and absence of latrunculin A. This was carried out by randomly selecting 50 events per condition (from 5 cells each) which had the characteristic of being clearly separable from any nearby events (i.e. rose from a clear baseline, and descended to a clear baseline). Events were matched so that pre- and post-stimulation events came from the same cells. From these events, we were able to distinguish clear fall times, which correspond to loss of vesicular content (see Fig. 5A). Events were aligned to the peak before the fall, and then averaged. These data are shown in for control cells, and latrunculin treated cells (Fig. 5B). Surprisingly, we found that there was a significant differences between the average t_1/2_ of decay between events before and after stimulation, in that those events occurring after stimulation showed faster expulsion of content (t_1/2_  = 1386 ms ± 27.4 compared to 511 ms ± 20.3, mean ± sem, n = 50, p<0.001). Pre-treatment with latrunculin A changed this relationship (Fig. 5C), greatly speeding the rate of expulsion in unstimulated cells (510 ms ± 21.5) while slowing the rate after stimulation (924 m s ± 24.6 mean+sem, n = 50, p<0.001). Treatment with Jasplakinolide (Fig. 5D) gave a result of (1206 ms ± 26.3 before stimulation, compared to 1283 ms + 28.1 after stimulation, n = 50).

### Vesicle fusion occurs in-between regions of β-actin enrichment

**Figure 6:**
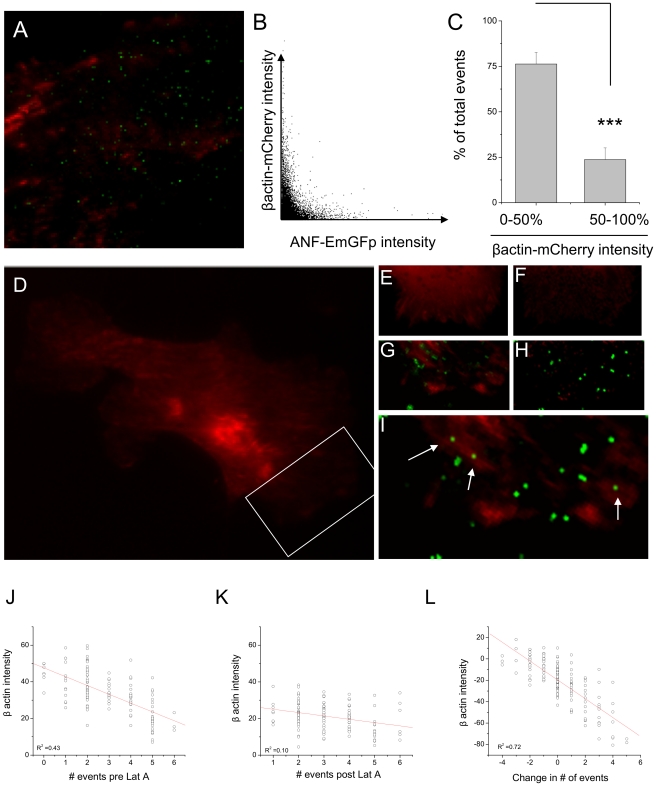
Separation between the actin cytoskeleton and exocytic site. (A) TIRFM image of β-actin -mCherry (red) overlaid with a summed intensity projection of the ANF-EmGFP image series. Note that green spots, corresponding to sites of secretion, do not align with regions of actin enrichment at the membrane. (B) Plot of summed GFP intensity against mCherry intensity, illustrating the negative relationship. (C) A third method of assessing the relationship between β-actin and site of secretion is to segment β-actin images into regions above and below 50% intensity, and count the number of events within each. Data are expressed as % of total events per cell, and are the average of 6 experiments. *** indicates p<0.001. (D) TIRFM image of a cell expressing β-actin -mCherry. Box indicates region of cell highlighted in B–F. (E) Wide field image of part of cell before and (F) after treatment with latrunculin, with equal image scaling. (G) TIRFM image of β-actin with summed GFP TIRF image, before and (H) after latrunculin treatment. (I) If the pre-latrunculin β-actin image is combined with the post-treatment image of secretion (summed ANF-EmGFP), it can be seen that secretion occurs at sites that were previously unavailable. (J) Pre-latrunculin intensity of β-actin labeling within ROIs used for secretory analysis, plotted against the number of events seen within those regions, showing a negative correlation. (K) this relationship disappears after latrunculin treatment. (L) Relative change in β-actin intensity before and after latrunculin treatment is plotted against change in number of events for the same regions.

If, as has been suggested [5], [6], actin plays a barrier role to secretion, it would be expected that sites of secretion should contain less actin than surrounding regions. Using two color TIRFM we visualized β-actin mCherry and sites of vesicle fusion at the plasma membrane, to examine the relationship between the two. Fig. 6A shows a TIRFM image of β-actin mCherry, with sites of fusion highlighted by a summed intensity projection of ANF-EmGFP. This illustrates the extent to which sites of secretion correspond to regions that lack actin enrichment at the membrane. This is further illustrated by a plot of β-actin mCherry pixel intensity against EmGFP pixel intensity for the summed image (Fig 6B), which shows an inverse correlation. As an alternative method, we have segmented the β-actin mCherry fluorescence for 6 cells into above and below the average intensity, and calculated the number of events in each region. Fig. 6C shows that the large majority of events occur in regions with reduced β-actin levels.

### Disruption of β-actin provides new sites of fusion competence

The barrier hypothesis for actin, raises the question of what happens at the membrane when the cytoskeleton is disrupted. To this end, we examined regions of the cell before and after latrunculin treatment. Fig. 6D shows an image of a cell expressing β-actin mCherry, prior to latrunculin treatment. The cell was co-transfected with ANF-EmGFP (not shown). Figs. 6E and 6F show a portion of the cell before and after latrunculin treatment. Figs. 6G and 6H show a comparison between the β-actin TIRF channel and summed ANF-EmGFP channel. In both cases, fusion occurred in-between sites of actin enrichment. Most interestingly, if the pre-latrunculin treatment β-actin channel is aligned with the post-latrunculin ANF-EmGFP channel, some sites of fusion are seen to appear which were previously blocked by actin (6I). If regions of interest are examined before and after latrunculin treatment, it can be seen that there is a weak correlation between the number of events per region, and the intensity of β-actin staining (Fig. 6J). This correlation disappears following latrunculin treatment (Fig. 6K). If the change in β-actin staining is plotted against the change in events pre region (Fig. 6L) a clear relationship can be seen.

## Discussion

The role of the actin cytoskeleton in secretion has long been controversial. While there has been no shortage of evidence supporting its involvement, it is the detailed mechanism of its role that remains unclear. Presynaptic terminals are enriched in actin and experiments in neuroendocrine cells have indicated that actin plays a barrier role in preventing excessive secretion [5], [6]. At the same time, other experiments have indicated a more important role in endocytosis and vesicle mobilization [3], [4], [20], [21].

Our initial finding was that actin depolymerization by Latrunculin A perturbed the kinetics of two phases of release of secretory vesicles, leading to an overall decrease in the amount of release observed following 2 minutes of stimulation (Fig. 3). These findings indicate that a portion of the actin cytoskeleton acts as a fusion clamp, while other elements provide a route for the delivery of vesicles to the membrane. These findings, plus the observation that Latrunculin A treatment provides an overall decrease in secretion, provide a picture of neuroendocrine secretion that is broadly similar to non-calcium dependent exocytosis described in acinar cells [22], where actin filament disassembly was necessary and sufficient for exocytosis, but a minimally intact actin cytoskeleton was also necessary for fusion.

### Vesicle delivery; a role for actin in the delivery of vesicles to a plasma membrane pool

While secretion can be followed in PC12 cells by tracking whole cell fluorescence, amperometry, or capacitance, TIRFM allows us to observe the fates of individual vesicles (e.g. refs [7], [8]) in a temporally and spatially distinct fashion. Using TIRFM, we were able to monitor the arrival of vesicles at the plasma membrane, and depolarization with 30 mM K+ promoted this process. In contrast to wide-field techniques, this provides information on a vesicle by vesicle basis, but with the restriction that only a very narrow portion (∼100 nm) of the cell is observed. Consequently, the only vesicles observed are those present in the very outer regions of the actin cortex. In chromaffin cells, this cortex is seen to be confined to a narrow ring around the plasma membrane [6], [11], [12]; however, in PC12 cells the actin cytoskeleton forms a more diffuse network (Fig. 1). Prior to stimulation, disruption of actin actually promotes fusion, as discussed above, consistent with previous work implicating calcium-dependent regulation of the actin cortex in secretion [23]–[24]. This involves both a reduction in the spatial specificity of release, and removal of a fusion break. However following stimulation, after an initial burst of secretion, Latrunculin A treated cells show a relative loss of secretion. This appears to indicate that random diffusion to the site of secretion is insufficient to support the rate of vesicle exocytosis in stimulated cells. Although we have not examined this possibility, it also suggests that vesicle mobilization is calcium dependent (or stimulation dependent in some other fashion, such as indirectly through some other effector).

### Spatial regulation of secretion

Analysis of large numbers of secretory events indicates that there are specific sites where fusion is ‘permitted’, and proportionately, large areas where fusion does not seem to occur, something first observed in neuroendocrine cells by Robinson and colleagues [1]. Interestingly, we observed that treatment with latrunculin, which disrupts the actin cytoskeleton by sequestering monomers, increased the number of available sites. Consistent with this, we found that the probability of secretion occurring at a given site was in inverse proportion to the density of β-actin -mCherry labeling. Further support for a causal relationship came from experiments where we monitored secretion under control conditions, then disrupted actin and monitored secretion again. This revealed that some areas which had previously been unavailable for secretion became accessible once the actin was removed (Fig. 6). The two most likely conclusion which can be gathered from this are either that (1) actin was preventing secretory vesicles from accessing those regions of membrane, or (2) that the cytoskeleton was involved in clustering of secretory components. Future work will address these issues. In pancreatic acinar cells, the large size of the fusing zymogen granules enabled direct visualization of the actin coating on the granules [25]. In this case, elimination of actin through latrunculin treatment led to a reduced latency for release, consistent with the findings in the present work.

### A proposed role for actin in secretion

**Figure 7:**
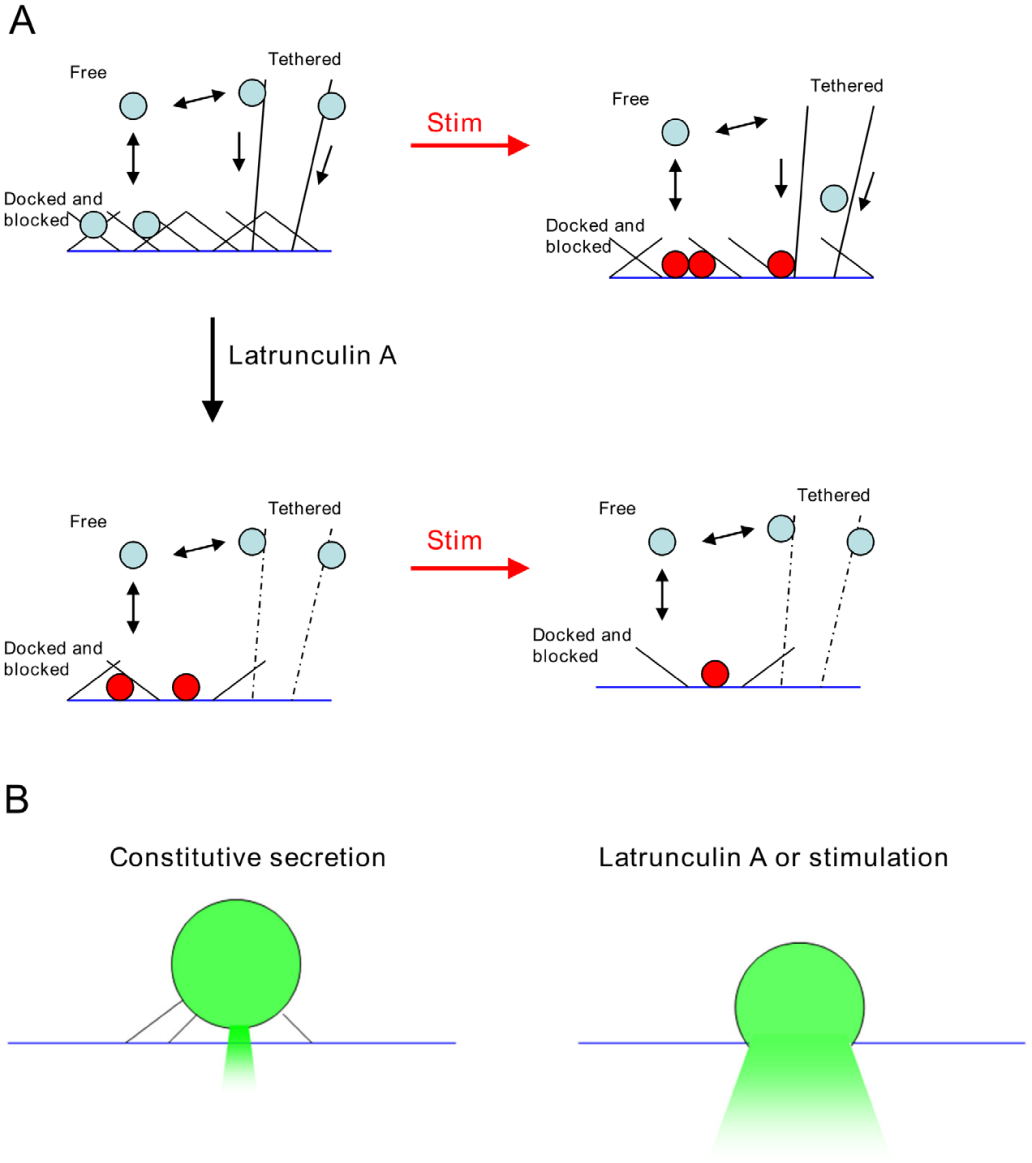
Models for the role of actin in secretion. (A) Role of actin in regulating availability of vesicle for secretion. At rest (top left) vesicles are in three possible pools; arrested by the cortical actin cytomatrix (“docked and blocked”) associated with actin filaments communicating with sites of secretion, and freely diffusing in the cytoplasm. On stimulation (top right) vesicles held by the cytomatrix become available for secretion, as do vesicles delivered by actin fibers. Latrunculin treatment (lower panel) disassembles much of the cytomatrix, allowing vesicles to spontaneously fuse at a higher rate, but impairing the ability of new vesicles to arrive at sites of secretion, due to loss of connecting actin filaments, leading to a reduced ability to sustain secretion following stimulation. (B) At the site of fusion itself, actin appears to slow the release of vesicular contents; this action is overcome by either latrunculin treatment or stimulation.

The evidence presented here indicates that actin plays a role in specifying release sites at the plasma membrane, preventing fusion in adjoining regions, while also being important for the delivery of vesicles for sustained secretion. This, together with the kinetic data for rates of sustained secretion (Fig. 3) or vesicle delivery (Fig. 4), allows us to propose a model for the role of actin in secretion (Fig. 7A). In this model, three groups of vesicles contribute to secretion; vesicles locally detained within the actin cortex (‘Docked and blocked’), vesicles freely diffusing at a moderate distance from the fusion site (‘free’), and vesicles attached to deeper actin filaments (‘tethered’). Stimulation causes release of vesicles in ‘Docked and blocked’ pool, and also promotes delivery of vesicles from the ‘Tethered’ pool. Latrunculin A treatment promotes fusion of vesicles in the ‘Docked and blocked’ pool, but converts the ‘Tethered’ vesicles to ‘Free’ ones, resulting in a weaker response to stimulation. At present, it is unclear exactly how vesicles are anchored to the cytoskeleton, although it has been proposed that synapsin might play a role in this process. Synapsin is an actin binding proteins found in nerve terminals, that regulates synaptic vesicle mobility [26] and colocalizes with actin during vesicle recycling [27]. Against this hypothesis, however, stand data showing that in a mouse triple synapsin knockout model, neither vesicle mobility nor secretion were impaired, although vesicle number was reduced [28].

In figure 7B we show separately for clarity, the proposed role of actin in exocytosis itself, promoting rapid loss of contents from the vesicle lumen. Expulsion of fluorescently labeled contents from dense cored vesicles has been examined previously. In an elegant study [29], Chow and colleagues showed that loss of contents was dependent on the nature of the fluorescent proteins expressed within the granule. Felmy [30] also showed this, and saw that co-expression of a β-actin mCherry construct (not the same one as used in this study) retarded secretion of some but not all fluorescent vesicle cargoes. In that study, Jasplakinolide was seen to speed the release of tPA, and Latrunculin A was seen to slow it slightly, but they did not compare content expulsion before and after stimulation, only after. The vesicular marker used in this study, ANF-EmGFP, was developed as a pre-peptide label that would be hydrolyzed within the granule to release the EmGFP attached to only a short peptide (15), and so content expulsion times seen in the present study more closely match those observed for Vamp-pHluorin in the Felmy study (release or dequenching in a few frames). The role played by actin in regulating vesicular content expulsion may reflect conversion from kiss-and-run to full fusion [11], [31]–[34] or may reflect some other form of post-fusional regulation of content release [35], [36].

The work presented here was carried out in PC12 cells. Some aspects of dense cored vesicle fusion are likely to show variations due to specific cell type; for example pancreatic acinar cells have granules of 5–10 times the size of those seen in PC12 cells [25]. Even closely related chromaffin cells (from which PC12 cells were derived) have a very different distribution of actin, at least following dissociation (5,6,11). Consequently, the details of our model will not necessarily pertain to other cell types. Nonetheless, we believe the present work will be a valuable point of comparison, not least because it is in general consistent with what has been seen in other cell types. Our overall hypothesis derived from the work presented in here, is that the actin cytoskeleton plays four roles in secretion. (1) a role in specifying release site, (2) a role in tethering membrane proximal vesicles, (3) a role in delivering ‘new’ vesicles from deeper within the cell, and (4) a role in regulating fusion pore properties.

## Materials and Methods

### Cell culture

PC12 cells (ATCC) were maintained in flasks in F12 medium (Invitrogen) supplemented with 15% horse serum (ATCC) and 2.5% Fetal bovine Serum (Invitrogen). Prior to experiments they were split, transfected, and seeded into 6 well dishes containing acid-washed, cover slips previously coated with GelTrex (Invitrogen). Transfections were carried out using electroporation in a Gene Pulser (Bio Rad) using 20 µg DNA.

ANFP-EmGFP was a generous gift from Dr Ed Levitan, University of Pittsburgh. β-actin (actb gene from *H. sapiens*) was obtained as full length cDNA from Origene, and subcloned into the pmCherry plasmid from Clontech. All constructs were fully sequenced to ensure accuracy.

### Imaging

Cells were imaged 3 days after transfection, in saline consisting of 140 mM NaCl, 5 mM KCl, 2 mM CaCl2, 2 mM MgCl2, 5.5 mM glucose, 20 mM HEPES buffered to pH 7.3 using NaOH. Cover slips with cells were mounted in an imaging chamber (Warner Instruments) and viewed from beneath with conventional epifluorescence optics. Image acquisition was controlled by a computer running Slidebook 4.2 (Intelligent Imaging Innovations). Imaging was carried out on an Olympus ix80 inverted microscope with integrated high precision focus drive. Fluorescence excitation was provided by a rapid switching DG4 light source (Sutter Instruments) attached by liquid light guide. The cooled CCD camera was a Hammamatsu ORCA R2, communicating to the host computer via firewire interface. For widefield illumination timelapse experiments, a 40X oil immersion objective was used (Olympus UPlanSApo 40×0.95 NA), while for 3D or TIRFM experiments, we used an APO N 65×1.49 NA objective optimized for fluorescence (Olympus). For 3D imaging, the optical sections were taken at 200 nm intervals. Digital deconvolution within Slidebook was carried out using point-spread functions empirically derived on the microscope using Point Speck beads from Molecular Probes. 3D reconstructions were carried out using SoftWorX suite from Applied Precision. TIRF illumination was achieved through the objective, using 488 and 536 nm lasers (Melles Griot), with fine adjustment by micrometers to optimize TIRF illumination. For immunocytochemistry experiments, cells were fixed in 4% paraformaldehyde and permeabilized with 0.5% Igepal in sterilized Phosphate Buffered Saline. Anti-PI(4,5)P2 and Anti- PI(3,4,5)P3 mouse primary antibodies (Echelon) were applied at 10 µL/mL PBS, and a secondary Alexafluor rabbit-anti-mouse secondary antibody was applied at 1 µg/mL.

### Image analysis

For widefield experiments, we measured a background region, and subtracted this from each frame. Each cell was then outlined and fluorescence measured over time to provide a timecourse of release following addition of KCl which brought the K+ concentration to 30 mM.

TIRFM experiments depend upon a near-zero background. For that reason we generated a minimum intensity projection for each cell, and subtracted this image from each frame. This subtracted any permanent features of the image from the stack, allowing arriving vesicles to be clearly seen. As this was a flat numerical subtraction, it did not affect vesicle rise and fall times. A maximum intensity projection image of this new stack was then used to identify regions of exocytosis, and each spot was surrounded by a 9 pixel region of interest (ROI). This ROI was then extracted as an average intensity over time plot, and analyzed for peaks using Microcal Origin. Number and amplitude of peaks were then compared between conditions.
